# Increases of Chamber Height and Base Diameter Have Contrasting Effects on Grazing Rate of Two Cladoceran Species: Implications for Microcosm Studies

**DOI:** 10.1371/journal.pone.0135786

**Published:** 2015-08-14

**Authors:** Ying Pan, Yunshu Zhang, Yan Peng, Qinghua Zhao, Shucun Sun

**Affiliations:** 1 Department of Biology, Nanjing University, Nanjing, 210093, China; 2 School of Ecology and Environmental Sciences, Yunnan University, Kunming, 650031, China; 3 Key Laboratory of Mountain Ecological Restoration and Bioresource Utilization of Sichuan Province, Chengdu Institute of Biology, Chinese Academy of Sciences, Chengdu, 610041, China; 4 Department of Surveying and Mapping Engineering, Changjiang Institute of Technology, 9 Wenhua Road, Jiaxia District, Wuhan, 430212, China; University of Shiga Prefecture, JAPAN

## Abstract

Aquatic microcosm studies often increase either chamber height or base diameter (to increase water volume) to test spatial ecology theories such as “scale” effects on ecological processes, but it is unclear whether the increase of chamber height or base diameter have the same effect on the processes, i.e., whether the effect of the shape of three-dimensional spaces is significant. We orthogonally manipulated chamber height and base diameter and determined swimming activity, average swimming velocity and grazing rates of the cladocerans *Daphnia magna* and *Moina micrura* (on two algae *Scenedesmus quadricauda* and *Chlorella vulgaris*; leading to four aquatic algae-cladoceran systems in total) under different microcosm conditions. Across all the four aquatic systems, increasing chamber height at a given base diameter significantly decreased the duration and velocity of horizontal swimming, and it tended to increase the duration but decrease the velocity of vertical swimming. These collectively led to decreases in both average swimming velocity and grazing rate of the cladocerans in the tall chambers (at a given base diameter), in accordance with the positive relationship between average swimming velocity and grazing rate. In contrast, an increase of base diameter at a given chamber height showed contrasting effects on the above parameters. Consistently, at a given chamber volume increasing ratio of chamber height to base diameter decreased the average swimming velocity and grazing rate across all the aquatic systems. In general, increasing chamber depth and base diameter may exert contrasting effects on zooplankton behavior and thus phytoplankton-zooplankton interactions. We suggest that spatial shape plays an important role in determining ecological process and thus should be considered in a theoretical framework of spatial ecology and also the physical setting of aquatic microcosm experiments.

## Introduction

Small-scale experiments such as microcosm and mesocosm studies are an important approach to generate and test ecological theories [1,2]. The microcosm approach offers many advantages for ecological research, including controllable environmental factors, simplified community structure, rapidness, and repeatability, as well as being less costly compared with field studies [3]. Because microcosm experiments are often conducted at a small spatial scale and may distort important features of natural ecosystems [4,5], ecologists need to perform multiple-scale experiments by changing microcosm chamber volume to determine the effects of spatial scale on ecological processes [6,7]. Comparing experimental results between different microcosm volumes may reveal the scale effect on behavior, growth and reproduction, and population dynamics of phytoplankton-zooplankton systems [1,6,8]. For example, significant scale effects on zooplankton behavior have been found in studies with chamber volumes ranging from 10 mL to more than 500 mL [2,8–12]; similar effects have been observed in sex ratio and reproduction of the cladocaran species (*Moina micrura*) when the culture volume increased from 30 to 120 ml [13].

Importantly, increasing microcosm volume can be achieved by increasing either chamber height, or base area, or both. Among the previous studies addressing effects of spatial scale on phytoplankton-zooplankton interactions, some examined the effects of increasing chamber height [6], and some others examined the effect of base area [14] or both [1,5,13]. To the best of our knowledge, however, few studies have explicitly distinguished the effects of increasing chamber height and base area, and hence it is not clear whether increasing height has the same effect on the ecological processes as increasing base area, i.e., whether the effect of the shape of such a three-dimensional space on species interactions should be determined.

Indeed, increasing chamber height and base area may exert differential ‘scale’ effects on aquatic ecological processes that are commonly addressed in phytoplankton-zooplankton interactions. First, from a biomechanical perspective, chamber height and base area may have different effects on swimming activity and grazing rate of zooplankton grazers. Many zooplankton species (e.g. cladocerans) are greater in specific gravity than water, and therefore they have to spend more energy to travel upward than individuals travelling the same distance horizontally. Moreover, when Reynolds numbers (the ratio of inertial force [the difference between gravity and buoyancy] to viscous force) are greater than 1 in the cladocerans [15], they will spend more than twice the energy to swim upward (against both gravity and viscous drag) for the same distance than horizontally (against viscous drag only). Hence, even if swimming downward is free of energy cost, vertical swimming will cost more than horizontal swimming for a given distance. According to the random walk hypothesis of cladocerans in homogeneous environments [16,17], the swimming frequency along vertical or horizontal direction for the organisms living in a chamber should be proportional to the maximum trajectory length along the vertical or horizontal direction (i.e. chamber height or base diameter). Thus, cladocerans are expected to swim vertically more often than horizontally in taller chambers (at a given base area) and therefore have to expend more energy and/or time to obtain a similar level of food (resulting in a lower grazing rate).

Increasing chamber height and base area may have also different effects on gas exchange for cladoceran growth and reproduction. For example, for a given volume deep waters with small air-water interface area are usually characterized by lower concentration of dissolved oxygen (DO) than shallow waters [18–20]. Low DO concentration may have direct negative effects on the behavior, growth, reproduction, and grazing efficiency of cladocearn species [21,22].

Accordingly, increasing chamber height alone may not only impede the processes of gas exchange between air and water, but also increase energy costs to overcome gravity in vertical swimming (especially when Reynolds numbers > 1), which would decrease the swimming ability of cladocerans. In contrast, increasing base area at a given height will likely increase their swimming ability. In addition, numerous studies have shown that grazing rates (on phytoplankton) largely depends on swimming ability in cladocerans [23,24]. We thus can hypothesize that increases of chamber height and base area, i.e. the change in microcosm shape, would have opposite effects on swimming ability and grazing rate of cladocerans.

**Fig 1:**
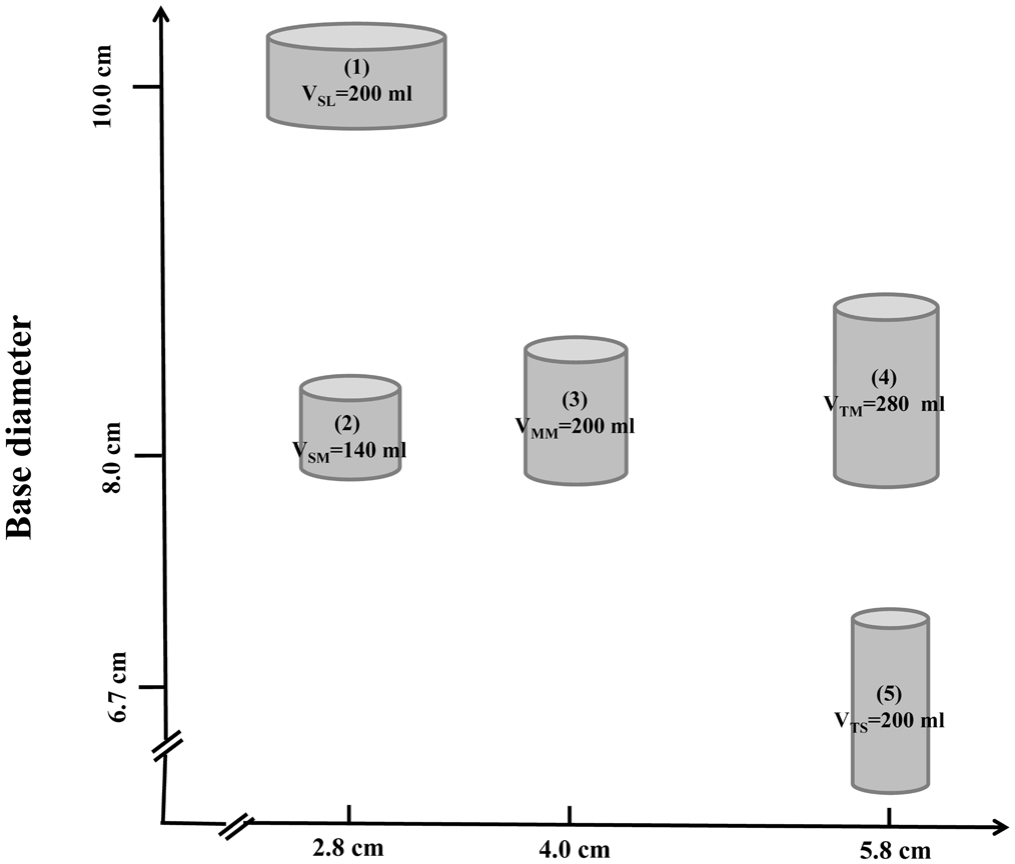
Illustration of the experimental design showing five different chamber treatments. The experiment included four algae-grazer systems including *Scenedesmus quadricauda*-*Daphnia magna*, *S*. *quadricauda*-*Moina micrura*, *Chlorella vulgaris*-*D*. *magna*, and *C*. *vulgaris*-*M*. *micrura* system, and each system was subject to five treatments incorporating chamber height and base diameter as treatment factors. SL: short height and large diameter; SM: short height and medium diameter; MM: medium height and medium diameter; TM: tall height and medium diameter; TS: tall height and small diameter.

To test this hypothesis, we conducted a microcosm experiment, in which we orthogonally manipulated both chamber height and base diameter (see Fig 1) and examined the responses of two cladoceran species (*Daphnia magna* and *Moina micrura*) in terms of their swimming activity, average swimming velocity and grazing rates on two phytoplankton species (*Scenedesmus quadricauda* and *Chlorella vulgaris*), respectively. Because Reynolds numbers of the individuals of the study species used were greater than 1, we predicted that: 1) increasing chamber height would lead to decreases in average swimming velocity and grazing rate at a given base diameter, 2) increasing base diameter would lead to increases in average swimming velocity and grazing rate at a given chamber height, and 3) at a given chamber volume, the average swimming velocity and grazing rate would decrease with increasing ratio of chamber height to base diameter.

## Methods

### Experimental organisms

Two algal and two cladoceran species were used in our experiment. The two algal species were *Scenedesmus quadricauda* (FACHB-1297) and *Chlorella vulgaris* (FACHB-8). Both algal species samples were obtained from the Freshwater Algae Culture Collection of Institute of Hydrobiology, the Chinese Academy of Sciences. Both species can shift from a unicellular to a colonial state depending on abiotic (e.g. nutrient enrichment) and biotic (e.g. exposed to predator) factors [12]. Single cells of *S*. *quadricauda* are oval in shape with the average length being 25 μm and the width 18 μm, while single cells of *C*. *vulgaris* are spherical in shape with the diameter being 5–10 μm. Algae samples were cultured separately and aseptically in COMBO medium in an incubator at 20°C with a light–dark cycle of 14:10 and a light intensity of 50 μmol photons m^-2^ s^-1^. The cultures were maintained in exponential growth phase by regular dilution with fresh medium.

The two cladocerans were *Daphnia magna* and *Moina micrura* and both species were isolated from Lake Taihu, East China and were among the dominant species that are closely coupled with phytoplankton biomass in aquatic environments. Adult *D*. *magna* are 2.3–6.0 mm in body length, much larger than *M*. *micrura* (0.5–1.3mm) [25,26] and thus are potentially more efficient in consuming large food particles [27]. We cultivated monoclonal groups of the two grazer species in COMBO medium, and fed them with either *S*. *quadricauda* or *C*. *vulgaris* at a rate of 10^5^ cells mL^-1^ per day for 3 months prior to the experiment, resulting in four algae-grazer systems in total, including 1) *S*. *quadricauda* and *D*. *magna*, 2) *S*. *quadricauda* and *M*. *micrura*, 3) *C*. *vulgaris* and *D*. *magna*, and 4) *C*. *vulgaris* and *M*. *micrura*. This research was conducted under Law of the People’s Republic of China on the Protection of Wildlife (August 28, 2004). No permits were required to carry out this study. All animal work was approved by the Animal Care Committee at Nanjing University.

Prior to experiment, clones of the two grazer species (2 to 3 days old), which were collected from beakers in clean COMBO medium, were starved for 4 h so that their guts were cleared of pre-fed food. Only medium-sized individuals were used in the experiment for each grazer species (about 1.5 mm long for *D*. *magna* and 0.85 mm long for *M*. *micrura*), referring to Ghadouani et al. [11], because large individuals might bear embryos and reproduce during experiments and small individuals were hard to trace during behavioral observations. Reynolds numbers of *D*. *magna* and *M*. *micrura* ranged from 2.58 to 3.73 and from 2.06 to 2.89, respectively, based on the formula that Reynolds numbers = *UL*/*v*, Where *U* is the three-dimensional instantaneous velocity (ranging from 0.173 to 0.252 cm s^-1^ and from 0.244 to 0.342 cm s ^-1^ for *D*. *magna* and *M*. *micrura*, respectively; calculated from the results of the current study), cm s^-1^; *L* is the body length, cm; and *v* is the coefficient of kinematic viscosity, cm^2^ s^-1^ [28].

### Experimental design

The experiment was conducted in a laboratory at Nanjing University. The experiment had five chamber treatments (using cylindrical beakers): 1) short height (2.8 cm) and large diameter (10 cm; 200 mL in volume), namely SL, 2) short height (2.8 cm) and medium diameter (8.0 cm; 140 mL in volume), namely SM, 3) medium height (4 cm) and medium diameter (8.0 cm; 200 mL in volume), namely MM, 4) tall height (5.8 cm) and medium diameter (8.0 cm, 280 mL), namely TM, and 5) tall height (5.8 cm) and small diameter (6.7 cm, 200 mL), namely TS. The experimental treatments were schematically presented in Fig 1. We set our experimental volume at a small scale to presumably minimize physico-chemical differences across the water column. Nevertheless, the volume was within the range that was used in many previous studies addressing zooplankton activity and phytoplankton-zooplankton interactions, e.g., from 30 ml to 120 in Martínez-Jerónimo et al. [13] and from 180 ml to 6400 L in Dodson et al. [8].

Comparisons between treatments 1) and 2) and between 4) and 5) could determine the effect of base diameter (at a given chamber height), comparisons among 2), 3), and 4) could determine the effect of chamber height (at a given base diameter), and comparisons among 1), 3) and 5) could determine the effect of chamber shape (the ratio of chamber height to base diameter) at a given volume on the swimming activity and grazing rate of the grazer individuals. The experiment design was applied to each of the four algae-grazer systems, resulting in 20 treatments, each treatment having 12 replicates (beakers). Furthermore, additional treatments including the algal species only (either *S*. *quadricauda* or *C*. *vulgaris*) were set as a parallel to the five chamber treatments, resulting in 10 treatments in total, such that the grazing rate of the two grazer species could be estimated. Each treatment had six replicates.

For each experiment containing grazers, grazer individuals were transferred into chambers (beakers) of specific treatments containing corresponding volumes of COMBO medium (see Fig 1), followed by introduction of concentrated inoculums of *S*. *quadricauda* or *C*. *vulgaris*, which were cultivated as noted above. Initial grazer densities in all treatments were 50 individuals L^-1^, which was lower than those in some previous studies addressing grazing rate, e.g., 400 individuals L^-1^ in Lürling [29] and Panosso and Lürling [30], since this was helpful to minimize the difficulty in behavior observations. Initial algal density was approximately 5×10^4^ cells mL^-1^, which was above the incipient limiting level for grazer species and has been used in many other microcosm experiments [31,32].

Half of the beakers that contained grazers were used to investigate the activity of the grazers. The other half of the beakers, together with the beakers containing one algal species only, were capped with breathable polyethylene films and would be used to investigate the grazing rate of the grazers. All the beakers were then transferred to oscillation incubators (growth chamber; TS-2102GZ, Shanghai Anjing laboratory equipment Co., Ltd) that were set at 20°C with light intensity being about 50 μmol photons m^-2^ s^-1^.

### Behavioral investigation

This investigation was conducted at 20°C in lab. Standard fluorescent bulbs were installed approximately 2 m away from and around (including the top and all sides) the experimental setup, providing homogeneous illumination (about 40 μmol photons m^-2^ s^-1^). Two video cameras (GZ-VX855BAC, JVC, Japan; spatial resolution: 1920×1080 pixels) were placed orthogonally at a distance of 25 cm from the central of the beaker bottom. This physical setting allowed for concurrent recording of the swimming activity of grazer individuals in both horizontal and vertical directions. After the beakers were mechanically stirred at 60 rpm for 5 minutes in oscillation incubators to homogenize the algal distribution in the beakers, the beakers were taken out the incubators and then the grazers were allowed to acclimate to the new environment for 15 minutes in the lab. Subsequently, the grazer activity was filmed at a rate of 60 frame s^-1^ for about 3 minutes for each replicate beaker and for each treatment.

Four types of activities were recorded for one randomly chosen grazer following previous studies [33,34]: horizontal swimming, upward swimming (swim vertically upward), downward swimming (swim vertically downward), and quiescent status. Video tapes were reviewed using an image measurement tool (Adobe After Effects CS4). We first recorded the time for the four types of activities, and then we chose the fragments that contained swimming trajectories away from the walls (in the middle of both views of the camera) of the chamber and were longer than 2 s. From the fragments, we calculated instantaneous swimming velocity [34,35] as the distance traveled by the grazer individual between two frames (i.e. 16.9 ms) using ImageJ 1.46 and MTrackJ plugin [36,37]. While analyzing the instantaneous swimming velocity of an individual, we calibrated each video to convert pixels into real distances (cm) using reference marks that were made in each beaker. Finally, the average swimming velocity (*V*_average_, mm s^-1^) for this grazer individual was approximated as follows: *V*_*average*_ = (*V*_*h*_ *T*_*h*_ + *V*_*u*_ *T*_*u*_ + *V*_*d*_ *T*_*d*_)/ (*T*_*h*_ + *T*_*u*_ + *T*_*d*_ +*T*_*q*_), Where *T*_*h*_, *T*_*u*_, *T*_*d*_ and *T*_*q*_ were the durations of horizontal swimming, upward swimming, downward swimming and quiescence (s), respectively; *V*_*h*_, *V*_*u*_, and *V*_*d*_ were the velocities of horizontal swimming, upward swimming, and downward swimming (mm s^-1^).

Three grazer individuals were randomly chosen from each beaker to determine the above-mentioned parameters, and thus a total of 108 grazer individuals per plankton system (3 individuals per chamber × 6 chambers per treatment × 6 treatments) were followed in the behavioral experiment. Finally, the metrics of these grazer individuals were first averaged for each beaker and then for each treatment.

### Grazing rate

The beakers were mechanically stirred at 60 rpm for 5 minutes in oscillation incubators every 1 hour throughout the experiment to facilitate gas exchange and keep the algae in suspension. The algae were sampled 10 hours after the beginning of the experiment; the grazing duration was longer than in some previous studies because of the low grazer density in our experiment [29,30]. At the end of the experiment, algal densities in all treatments were greater than 3.9 × 10^4^ cells mL^-1^, indicating that the grazing rate of the cladocerans was unlikely to be food-limited throughout the grazing experiment [31,32].

During sampling, we first measured the concentration of DO using a Hach HQ 40 d oxygen probe (Hach, Loveland, Colorado, USA) immediately after the beakers were taken out of the incubator. Then, 2 mL of the solution was removed to a 10 mL tube that contained 0.1 mL of Lugol’s preservative for microscopic enumeration of algae cells. Algal density was directly determined using an inverted light-microscope (Nikon Eclipse E100 microscope with a DS-2Mv-L2 camera, Nikon Corp., Tokyo, Japan) at the × 200 or × 400 magnification. The grazing rate (*G*, mL animal individual^-1^ h^-1^, i.e., clearance rate) was calculated as the difference in algal density between the experimental treatments (with grazer) and the corresponding controls (without grazers) according to the commonly used equation for plankton [38]: *G* = *V* [In (*C*_*0*_)- In (*C*_*1*_)] / *Nt*, Where *V* is the volume of culture (mL); *C*_*0*_ and *C*_*1*_ are the algal density at the end of the experiment in the control and experimental chambers (cell mL^-1^), respectively; *N* is the grazer number (individuals) and *t* is the experimental duration (10 h).

### Data analyses

Three-way ANOVAs were used to determine effects of one of the scale factors (chamber height at a given base diameter, base diameter at a given height, or chamber shape at a given volume), grazer species, and algal species on average swimming velocity and grazing rate for the whole dataset pooled from the experiments with four different algae-grazer systems. For single experiments with a specific system, one-way ANOVAs were used to determine the effect of scale factors on DO concentration, swimming activity, average swimming velocity, and grazing rate of the grazer. Once significant effects were detected, Tukey's *post hoc* tests were used to determine the difference between treatments. Linear regression analyses were conducted to determine the relationship between chamber shape and the duration and velocity of each swimming activity (including quiescent status, horizontal swimming, upward swimming, and downward swimming), and the time ratio of vertical to horizontal swimming during observations, and between average swimming velocity and grazing rate pooled from the chamber treatments for each algae-grazer system. Analyses were carried out using IBM SPSS19.0 package (SPSS Inc., USA). All data in tables and figures are presented as means ± s. d.

## Results

### Dissolved oxygen (DO) concentration

During the experiment, DO was unaffected by chamber height, base diameter and chamber shape (all P > 0.05), and was always higher than 7.9 mg L^-1^ in all the treatments during the experiment (S1 Table), indicating that oxygen availability was not a potential limitation for the growth of grazer individuals.

### Swimming activity and average swimming velocity

**Fig 2:**
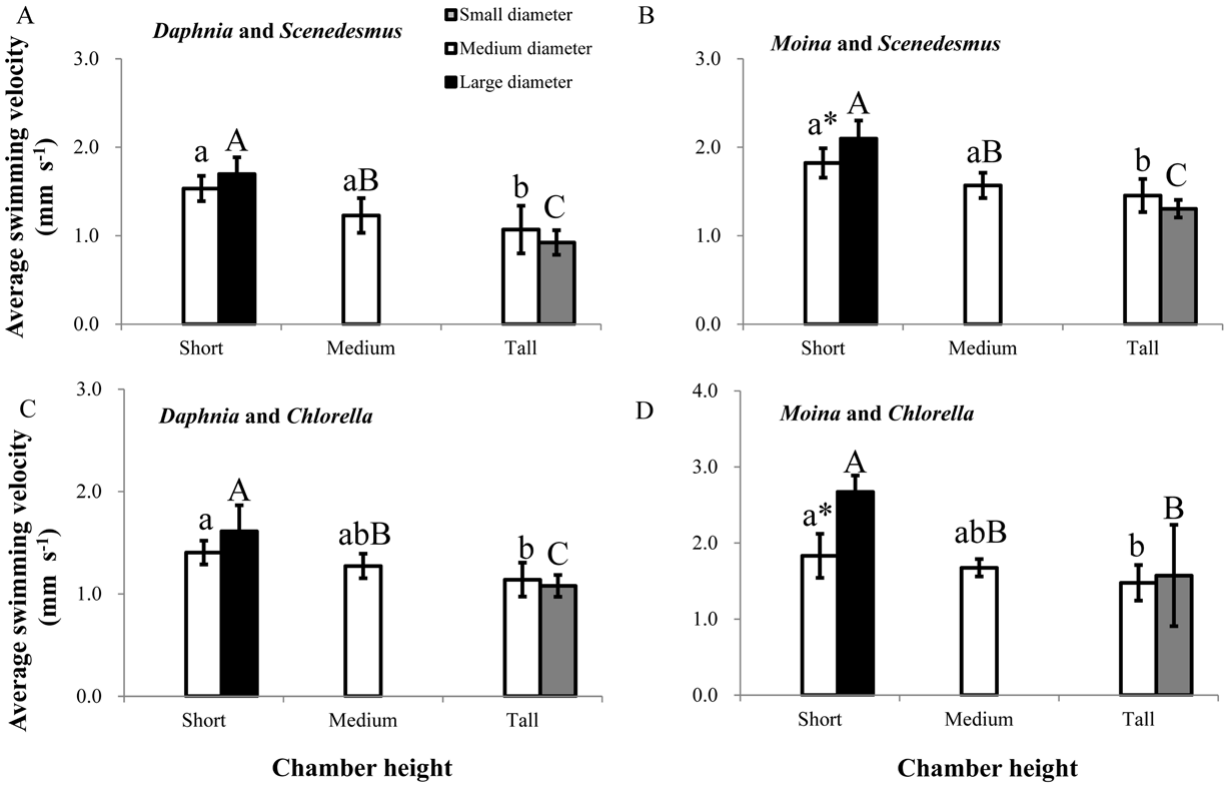
Average swimming velocity (means ± s. d, n = 6) of grazer species in different plankton systems under three levels of chamber height and three levels of base diameter. (A) *Daphnia magna* fed with *Scenedesmus quadricauda*, (B) *Moina micrura*fed with *S*. *quadricauda*, (C) *D*. *magna* fed with *Chlorella vulgaris*, (D) *M*. *micrura* fed with *C*. *vulgaris*. Different lowercase letters indicate significant chamber height effect among treatments at a given base diameter. Different upper case letters indicate significant chamber shape effect at a given chamber volume. “*” indicates a significant difference between two treatments at a given chamber height.

In both *D*. *magna* and *M*. *micrura*, swimming activity and average swimming velocity were significantly affected by the scale factors (chamber height, or base diameter, or chamber shape) regardless of what algal species were fed (Table 1 and Table 2). At a given base diameter, increasing chamber height led to increases in the durations of quiescence and upward swimming, but decreased the velocity and duration of horizontal swimming (but generally non-significant for vertical swimming); therefore, average swimming velocity was reduced (P < 0.05, Fig 2). In contrast, at a given chamber height, increasing base diameter generally led to decreases in the durations of quiescence and upward swimming, but increased the velocity and duration of horizontal swimming; therefore, average swimming velocity was increased, especially for *M*. *micrura* individuals in short chambers (Fig 2B and 2D, P < 0.05). Furthermore, at a given chamber volume, with increasing ratio of chamber height to base diameter, the durations of quiescence and upward swimming increased, the duration and velocity of horizontal swimming decreased; therefore, the average swimming velocity decreased (all P < 0.05; Fig 2). Generally, the decreasing trend of average swimming velocity with increasing ratio of chamber height to base diameter was more obvious in *M*. *micrura* than in *D*. *magna* when using *C*. *vulgaris* as the exclusive diet (S2 and S3 Tables).

**Table 1:** Summary of three-way ANOVA determining effects of specific scale factor (height effect at medium base diameter, or base effect at a given chamber height, or shape effect at a given volume), grazer species, and algal species on average swimming velocity and grazing rate for the whole dataset pooled from all the experiments with four different systems containing grazers.

	Scale factor (S)		df	Algal species (A)	df	Grazer species (G)	df	S×A	S×G	A×G	S×A×G
Average swimming velocity (mm s^-1^)	Height effect	22.796***	2	0.216 ^ns^	1	68.190***	1	0.867 ^ns^	0.012 ^ns^	0.3512 ^ns^	0.355 ^ns^
	Base effect at short height	39.646***	1	2.434 ^ns^	1	84.724***	1	6.643*	9.965**	11.367**	4.897*
	Base effect at tall height	0.631^ns^	1	2.355 ^ns^	1	22.768***	1	0.948^ns^	0.203 ^ns^	0.041^ns^	0.216 ^ns^
	Shape effect	73.982***	2	7.698**	1	84.545***	1	0.552 ^ns^	0.689 ^ns^	1.974 ^ns^	2.130 ^ns^
Grazing rate (mL individual^-1^ h^-1^)	Height effect	31.055***	2	27.430***	1	11.049**	1	0.898 ^ns^	0.735 ^ns^	3.175 ^ns^	0.953 ^ns^
	Base effect at short height	38.308***	1	9.571**	1	2.804^ns^	1	0.323 ^ns^	1.781 ^ns^	0.319 ^ns^	0.777 ^ns^
	Base effect at tall height	16.075***	1	14.133**	1	11.281**	1	0.026 ^ns^	2.496 ^ns^	0.252 ^ns^	1.213 ^ns^
	Shape effect	214.468***	2	26.747***	1	10.395**	1	1.066 ^ns^	1.877 ^ns^	0.038 ^ns^	0.594 ^ns^

^ns^ P > 0.05

* P < 0.05

** P < 0.01

*** P < 0.001

**Table 2:** Swimming activity of *Daphnia magna* and *Moina micrura* in different algae-grazer systems under different microcosm conditions.

Plankton system		Time distribution (s)				Swimming velocity in specific directions (mm s^-1^)		
		Quiescence	Horizontal swimming	Upward swimming	Downward swimming	Horizontal swimming	Upward swimming	Downward swimming
***Scenedesmus-Daphnia***	SL	20±2.95C	106.09±9.48A	53.78±11.02C	20.74±1.17*A	2.32±0.33A	1.24±0.1A	1.34±0.36A
	SM	23.44±2.59b	95.9±10.15a	56.13±6.14b	24.92±3.1a	2.19±0.27a	1.19±0.11a	1.39±0.15a
	MM	30.71±4.31aB	78.33±7.94b B	75.78±15.49ab B	19.67±10.23a A	1.81±0.43ab B	1.06±0.2a AB	1.58±0.33a A
	TM	35.72±5.08a	65.96±16.12b	91.98±18.55a	23.47±11.8a	1.6±0.39b	0.94±0.21a	1.53±0.22a
	TS	37.85±4.98A	47.96±8.58*C	104.56±13.97A	22.08±5.41A	1.54±0.26B	0.89±0.08B	1.3±0.26A
***Scenedesmus-Moina***	SL	22.19±5.18*B	125.06±16.41A	33.92±3.35C	18.16±5.3B	2.72±0.39A	1.38±0.18A	1.96±0.36A
	SM	36.73±3.72b	108.06±11.15a	36.73±4.86b	21.9±7.65a	2.49±0.37a	1.61±0.18a	1.98±0.35a
	MM	43.9±7.14abA	95.09±12.03abB	42.42±1.97ab B	23.2±2.08a AB	2.32±0.36a AB	1.35±0.11b A	2.12±0.2aA
	TM	47.21±5.89a	90.81±9.1b	49.21±7.52a	20.79±10.63a	2.15±0.4a	1.33±0.21b	2.11±0.24a
	TS	49.69±4.87A	63.15±9.09*C	64.72±5.92* A	26.16±4.72A	1.89±0.22B	1.43±0.12A	2±0.24A
***Chlorella-Daphnia***	SL	23.25±3.33B	126.33±10.29*A	50.87±13.14C	18.33±6.41A	2.11±0.23A	1.32±0.22A	1.79±0.16A
	SM	26.58±4.96b	102.93±10.3a	60.43±14.9b	22.04±11.42a	1.89±0.28a	1.09±0.14a	1.7±0.18a
	MM	32.63±8.58abAB	92.93±11.08a B	66.79±11.69b B	21.74±17.88a A	1.69±0.25ab B	1.32±0.38a A	1.64±0.22a A
	TM	39.24±5.75a	70.68±9.57b	86.03±12.63a	19.16±7.52a	1.49±0.21b	1.29±0.2a	1.58±0.39a
	TS	42.43±8.76A	59.29±8.17C	93.32±7.34A	20.71±6.05A	1.34±0.21C	1.28±0.19A	1.68±0.2A
***Chlorella-Moina***	SL	19.2±4.07*B	157.46±19.24* A	18.44±5.37*C	10.69±11.72A	3.2±0.48*A	1.4±0.32A	2.36±0.4A
	SM	33.98±6.64a	120.89±31.05a	34.02±3.47b	16.02±18.24a	2.48±0.18a	1.32±0.21a	2.08±0.35a
	MM	39.64±4.34a A	109.49±15.37a B	42.97±5.86a B	17.52±21.25a A	2.26±0.19a B	1.47±0.28a A	2.17±0.24a A
	TM	44.3±9.39a	100.95±17.64a	44.31±4.47a	17.89±23.21a	2.14±0.34a	1.71±0.6a	1.94±0.27a
	TS	48.56±10.76A	82.22±20.25C	53.48±9.13A	21.89±24.13A	1.9±0.25 B	1.69±0.87A	2.39±1.02A

SL: short height and large diameter; SM: short height and medium diameter; MM: medium height and medium diameter; TM: tall height and medium diameter; TS: tall height and small diameter. Different lowercase letters indicate significant chamber height effect among treatments at a given base diameter. Different upper case letters indicate significant chamber shape effect at a given chamber volume.

“*” indicates a significant difference between two treatments at a given chamber height.

Data are presented as means ± s. d (n = 6).

Additionally, the ratio of chamber height to base diameter was significantly and positively associated with the duration of quiescence and upward swimming, and time ratio of vertical to horizontal swimming, respectively, but negatively associated with the duration of horizontal swimming, horizontal velocity, and average swimming velocity, respectively (all P < 0.05; S2 and S3 Tables).

### Grazing rate

**Fig 3:**
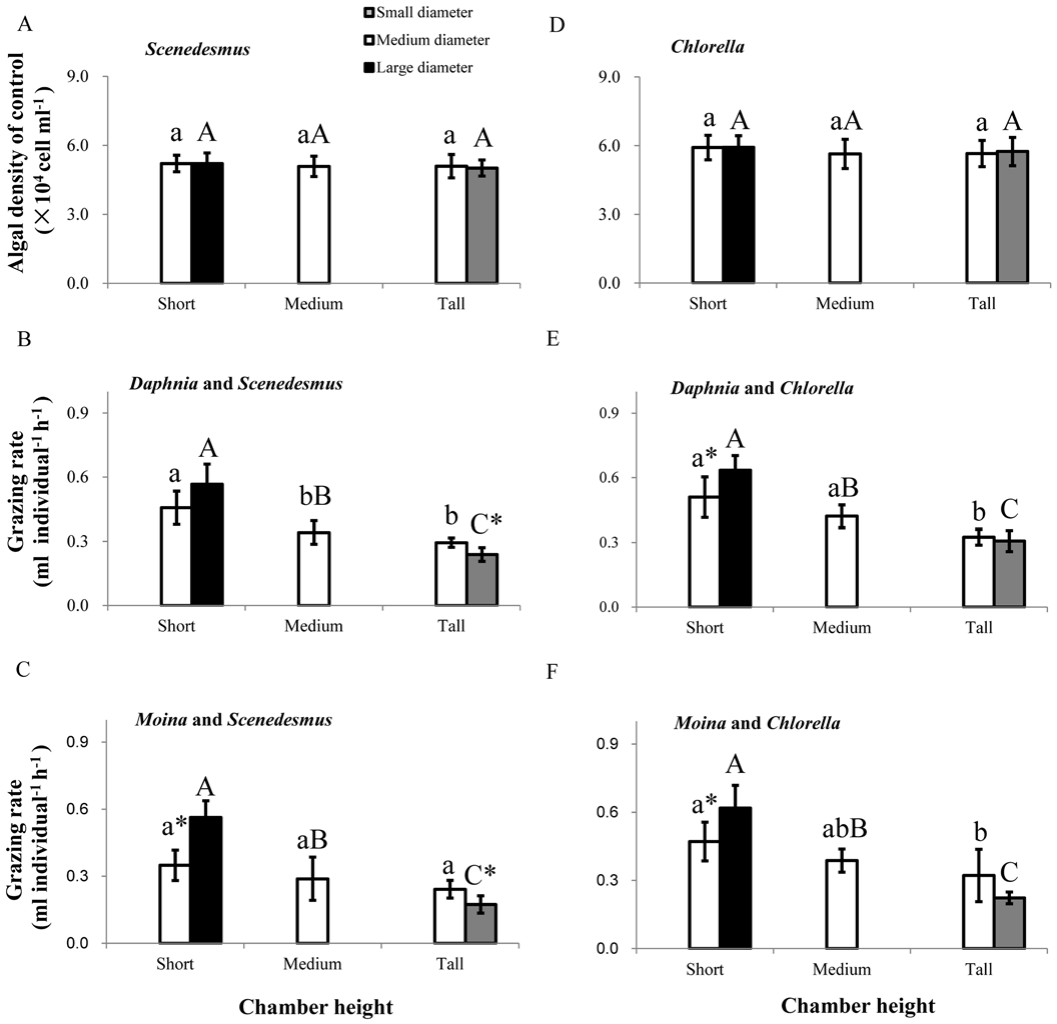
Algal density of *Scenedesmus quadricauda* and *Chlorella vulgaris* (control treatment), and the grazing rate of *Daphnia magna* and *Moina micrura* on these two algae. (A) Algal density of *S*.*quadricauda*; (B) grazing rate of *D*. *magna* on *S*. *quadricauda*, and (C) of *M*. *micrura* on *Scenedesmus*; (D) algal density of *Chlorella vulgaris*; (E) grazing rate of *D*. *magna* on *C*. *vulgaris*, and (F) of *M*. *micrura* on *C*. *vulgaris*. Different lowercase letters indicate significant chamber height effect among treatments at a given base diameter. Different upper case letters indicate significant chamber shape effect at a given chamber volume. “*” indicates a significant difference between two treatments at a given chamber height. Data are presented as means ± s.d (n = 6).

In the absence of grazers, algal density of *S*. *quadricauda* and *C*. *vulgaris* were unaffected by chamber height, base diameter and chamber shape (all P > 0.1; Fig 3). However, the effects on the algal density were significant in the presence of either of the grazers, indicating that the grazing rates were chamber shape-dependent (Fig 3). Specifically, at a given base diameter (medium diameter), the grazing rate of *D*. *magna* and *M*. *micrura* had undergone a steady decline with increasing chamber height. On the contrary, at a given chamber height, the grazing rates generally increased with increasing base diameter. Moreover, the grazing rates of both grazer species decreased with increasing ratio of chamber height to base diameter at a given chamber volume (P < 0.05), and the decreasing trend was generally more obvious in *M*. *micrura* than in *D*. *magna*, especially in systems with *C*. *vulgaris* as the algal species (S3 Table).

Both algal and grazer species identity significantly affected the grazing rate (Table 1). Generally, the grazing rates on *C*. *vulgaris* were significant higher than those on *S*. *Quadricauda* for both grazer species. *D*. *magna* was higher in grazing rate than *M*. *micrura* for both algal species.

**Fig 4:**
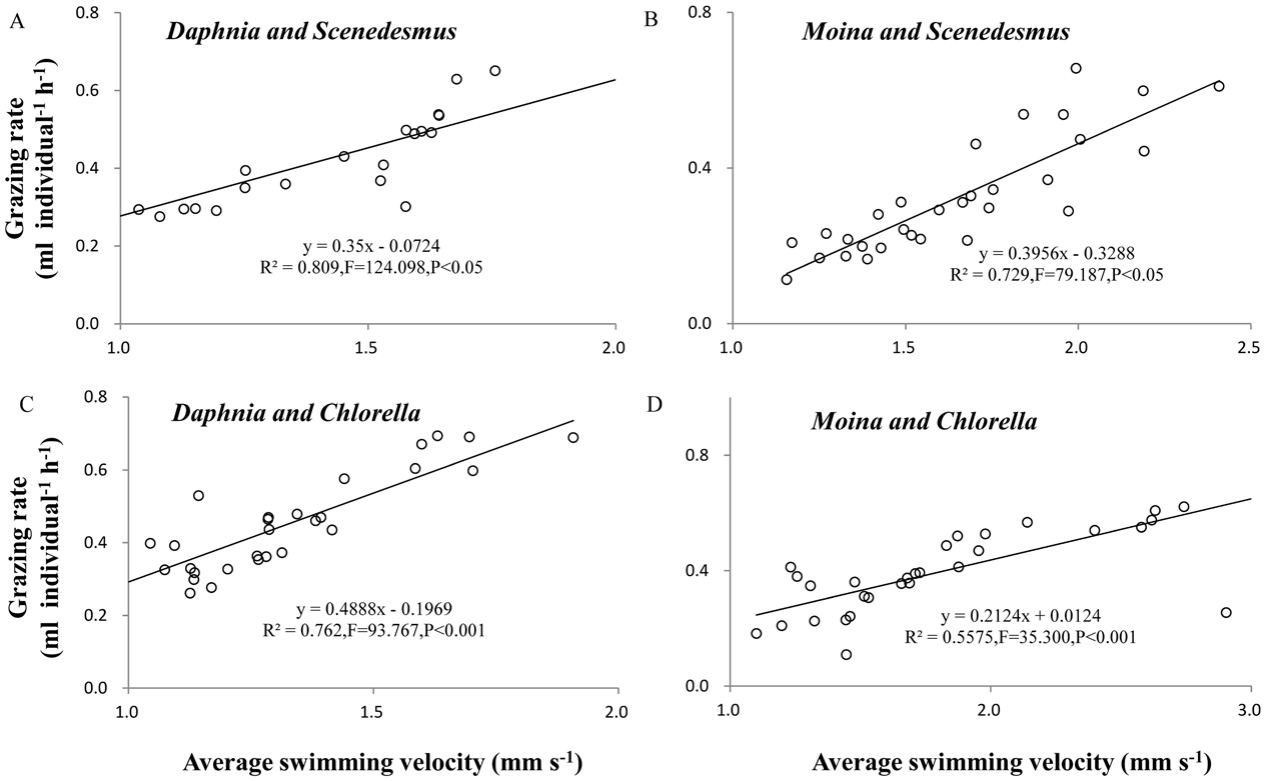
Linear regressions between average swimming velocity and grazing rate of grazer species in different algal-grazer systems. (A) *Scenedesmus quadricauda*-*Daphnia magna*, (B) *S*. *quadricauda*-*Moina micrura*, (C) *Chlorella vulgaris*-*D*. *magna*, and (D) *C*. *vulgaris*-*M*. *micrura* system.

In addition, the grazing rate significantly increased with increasing average swimming velocity in both *D*. *magna* and *M*. *micrura* across all the aquatic systems (Fig 4, R^2^ ranging between 0.55 and 0.81).

## Discussion

We have shown that increasing chamber height and base diameter have contrasting effects on average swimming velocity and grazing rate of the grazers in all the aquatic systems in our study. To the best of our knowledge, this is one of the few studies explicitly showing such contrasting ‘scale’ effects on ecological processes, although the effect of chamber height or base area have been individually noted in some previous microcosm studies [6,13]. Our results strongly indicate that the shape of chambers should be incorporated into microcosm studies testing the ‘scale’ effect on ecological processes.

The movement of zooplankton species is approximated as random within water columns, as reported in many cladoceran species, including *Daphnia* species [39,40] and *Ceriodaphnia dubia* [9] particularly in the absence of predators [17,24]. Such randomness should have occurred in the present study since the potentially confounding effects of age, predators and chemical tracers on the grazer mobility had been excluded [2,41]. As noted above, the height of the experimental chambers was relatively smaller than many other studies addressing algal-grazer relationships, which could minimize the differences in the algal density, temperature and in DO concentration between upper and lower water layers across water columns particularly when the experimental units were intermittently vibrated [20].

Given the randomness of grazer movement within the water column, increasing chamber height at a given base diameter means the increase in vertical path length of grazer swimming and the associated increase in duration in vertical length. As expected, with increasing chamber height at a given base diameter, the grazer did increase the duration of vertical swimming relative to that of the horizontal swimming, and moreover a reverse tendency was observed for the increase in base diameter at a given chamber height. More evidently, with increasing the ratio of chamber height to base diameter, the time ratio of vertical to horizontal swimming increased (S2 and S3 Tables).

The changed duration should have further affected swimming ability of zooplankters. Compared with horizontal swimming, travelers have to overcome both viscous drag and gravity when they swim upward [15]. Therefore, total mechanical energy expenditure is usually higher [42,43]. Probably because saving energy during grazing is crucial to survival and growth of grazers that are often limited in energy storage and expenditure [28], the swimming velocity of the grazers is lower in upward than horizontal swimming [42,44]. Likewise, many grazer individuals in this study and previous reports have been observed to sink to chamber bottoms or hover in the water column [44], possibly because they cannot offer the energy to keep swimming for a long time [45]. Thus, it is not surprising to observe that although the duration of the vertical swimming increased with increasing chamber height (at a given base diameter), the horizontal velocity was reduced and the duration of quiescence increased, which led to reduced average swimming velocity.

The grazing rate of cladoceran species has been well known to depend on the swimming velocity, assuming that the food capturing rate is positively associated with the encounter rate of a predator with its prey [23,24]. Usually more active grazers are more likely to encounter prey and thus may have a higher grazing rate. This velocity-grazing rate relationship was evident across the experimental units among treatments, in which the variation in average swimming velocity accounted for over 55% of the variation in the grazing rate for both grazer species, consistent with previous studies on the velocity-grazing rate relationship in *D*. *magna* [46]. Accordingly, the effect of chamber shape on average swimming velocity translated to the grazing rate of both grazer species.

In addition, the grazing rate varied with both the algal and grazer species identity. The large-bodied grazer species (*D*. *magna*) consumed more than the smaller one (*M*. *micrura*) and the grazing rate on the large-celled algal species (*S*. *quadricauda*) was smaller in both grazers. These findings are consistent with previous studies indicating that large grazers feed more efficiently than small ones to meet their greater energy demand for normal metabolism and growth [47], and large algae or colonies are often less palatable for filter-feeding cladocerans due to lower digestibility and because they are more mechanically obstructive [48]. Importantly, the chamber shape effect on grazing rate differed between the two grazer species regardless of what algal species they were fed; this implied that chamber shape might change the outcome of the interspecific competition for the algae between grazers.

In summary, we have shown that increasing chamber height and diameter may have contrasting effects on phytoplankton-zooplankton relationships, although such a shape effect is often negligible on microbial batch growth (due to Reynolds numbers << 1 for microbe) [49,50]. This result clearly suggests that spatial shape may significantly affect species interactions, which might have implications for natural ecosystems. Spatial shapes of habitats are diverse in nature: two-dimensional terrestrial habitats (e.g. farm fields and grasslands) may be circular or quadratic while being equal in size, and three-dimensional aquatic habitats (lakes and pools, and the present study) may be deep and narrow or shallow and broad even if the area or volume is constant. It is possible that variation in spatial shape in large-sale habitats would affect species performance and interspecific interactions for large-grained animals. Our result also has important implications for microcosm studies—a commonly used tool to generate and test ecological theories. As the chamber shape may significantly affect phytoplankton-zooplankton dynamics, future microcosm studies should not only consider the volume effect but also the shape effect on ecological processes. It is also worthwhile to note that we have provided a mechanistic understanding about the adaption of species to varied shapes (e.g. changed swimming velocity and duration) albeit at a small experimental volume. We call for more research into such shape effects on various ecological processes at different spatial scales.

## Supporting Information

S1 Table Dissolved oxygen concentration (means ± s. d, n = 6) in different plankton systems under different microcosm conditions. SL: short height and large diameter; SM: short height and medium diameter; MM: medium height and medium diameter; TM: tall height and medium diameter; TS: tall height and small diameter. No significant difference in the concentration was found among treatments at the 0.05 significance level.
(DOCX)

S2 Table Linear regressions between the ratio of chamber height to base diameter and specific swimming activity in two *Scenedesmus quadricauda*-grazer systems. (DOCX)

S3 Table Linear regressions between the ratio of chamber height to base diameter and specific swimming activity in two *Chlorella vulgaris*-grazer systems. (DOCX)
