# Supplementary material for: Increases of Chamber Height and Base Diameter Have Contrasting Effects on Grazing Rate of Two Cladoceran Species: Implications for Microcosm Studies
Source: PLoS One. 2015 Aug 14;10(8):e0135786. doi: 10.1371/journal.pone.0135786 (PMC4537195; doi:10.1371/journal.pone.0135786)
Supplement: S1 Table — SL: short height and large diameter; SM: short height and medium diameter; MM: medium height and medium diameter; TM: tall height and medium diameter; TS: tall height and small diameter. No significant difference in the concentration was found among treatments at the 0.05 significance level. (DOCX) [file pone.0135786.s001.docx]

**Table S1** Dissolved oxygen concentration (means ± s.d., n=6) in different plankton systems under different microcosm conditions. SL: short height and large diameter; SM: short height and medium diameter; MM: medium height and medium diameter; TM: tall height and medium diameter; TS: tall height and small diameter. No significant difference in the concentration was found among treatments at the 0.05 significance level.

| **Plankton system** | **n** | **Dissolve oxygen content (mg L^-1^)** | | | | |
| --- | --- | --- | --- | --- | --- | --- |
|  |  | **SL** | **SM** | **MM** | **TM** | **TS** |
| ***Scenedesmus*** | 6 | 8.00±0.31 | 8.11±0.29 | 8.02±0.27 | 8.02±0.21 | 7.95±0.20 |
| ***Scenedesmus* - *Daphnia*** | 6 | 8.08±0.31 | 8.09±0.23 | 7.98±0.26 | 8.08±0.24 | 8.06±0.26 |
| ***Scenedesmus* - *Moina*** | 6 | 8.10±0.41 | 8.07±0.36 | 8.05±0.24 | 8.02±0.31 | 7.96±0.21 |
| ***Chlorella*** | 6 | 8.08±0.24 | 8.07±0.25 | 7.96±0.24 | 8.00±0.33 | 8.03±0.28 |
| ***Chlorella* - *Daphnia*** | 6 | 8.10±0.18 | 8.04±0.11 | 8.00±0.28 | 8.01±0.29 | 7.96±0.25 |
| ***Chlorella* - *Moina*** | 6 | 8.08±0.32 | 8.08±0.30 | 8.00±0.25 | 8.03±0.24 | 8.05±0.17 |
