# Supplementary material for: Increases of Chamber Height and Base Diameter Have Contrasting Effects on Grazing Rate of Two Cladoceran Species: Implications for Microcosm Studies
Source: PLoS One. 2015 Aug 14;10(8):e0135786. doi: 10.1371/journal.pone.0135786 (PMC4537195; doi:10.1371/journal.pone.0135786)
Supplement: S2 Table — (DOCX) [file pone.0135786.s002.docx]

**Table S2** Linear regressions between the ratio of chamber height to base diameter and specific swimming activity in two *Scenedesmus* *quadricauda-*grazer systems.

|  |  | **n** | **df** | **Regression equation** | **R^2^** | **F** | **P** |
| --- | --- | --- | --- | --- | --- | --- | --- |
| ***Scenedesmus* - *Daphnia*** | Duration of quiescence (s) | 30 | (1,28) | y = 30.317x + 13.047 | 0.7341 | 77.324 | <0.001 |
|  | Duration of horizontal swimming (s) | 30 | (1,28) | y = -92.652x + 129.26 | 0.7968 | 109.801 | <0.001 |
|  | Duration of upward swimming (s) | 30 | (1,28) | y = 88.83x + 28.107 | 0.7068 | 67.498 | <0.001 |
|  | Duration of downward swimming (s) | 30 | (1,28) | y = 0.644x + 21.825 | 0.0004 | 0.011 | 0.916 |
|  | Time ratio of vertical to horizontal swimming | 30 | (1,28) | y = 3.2495x - 0.2 | 0.7997 | 111.797 | <0.001 |
|  | Horizontal velocity (mm s^-1^) | 30 | (1,28) | y = -1.3531x + 2.6293 | 0.4634 | 24.138 | <0.001 |
|  | Upward velocity (mm s^-1^) | 30 | (1,28) | y = -0.6065x + 1.3938 | 0.4853 | 26.849 | <0.001 |
|  | Downward velocity (mm s^-1^) | 30 | (1,28) | y = 0.0143x + 1.4196 | 0.0001 | 0.003 | 0.955 |
|  | Average swimming velocity (mm s^-1^) | 30 | (1,28) | y = -1.199x +1.7211 | 0.6717 | 56.678 | <0.001 |
|  | Grazing rate (mL individual^-1^ h^-1^) | 30 | (1,28) | y = -0.5007x + 0.6515 | 0.7229 | 73.204 | <0.001 |
| ***Scenedesmus* - *Moina*** | Duration of quiescence (s) | 30 | (1,28) | y = 39.056x + 18.692 | 0.6097 | 43.737 | <0.001 |
|  | Duration of horizontal swimming (s) | 30 | (1,28) | y = -87.502x + 144.05 | 0.7007 | 65.538 | <0.001 |
|  | Duration of upward swimming (s) | 30 | (1,28) | y = 47.869x + 19.353 | 0.7895 | 105.024 | <0.001 |
|  | Duration of downward swimming (s) | 30 | (1,28) | y = 8.5481x + 17.39 | 0.0802 | 2.442 | 0.129 |
|  | Time ratio of vertical to horizontal swimming | 30 | (1,28) | y = 1.4881x - 0.0204 | 0.6684 | 56.445 | <0.001 |
|  | Horizontal velocity (mm s^-1^) | 30 | (1,28) | y = -1.2454x + 2.9936 | 0.4148 | 19.789 | <0.001 |
|  | Upward velocity (mm s^-1^) | 30 | (1,28) | y = -0.1491x + 1.5015 | 0.034 | 0.987 | 0.329 |
|  | Downward velocity (mm s^-1^) | 30 | (1,28) | y = 0.1248x + 1.9642 | 0.0105 | 0.280 | 0.601 |
|  | Average swimming velocity (mm s^-1^) | 30 | (1,28) | y =-1.1323x + 2.0561 | 0.6713 | 56.255 | <0.001 |
|  | Grazing rate (mL individual^-1^ h^-1^) | 30 | (1,28) | y = -0.5226x + 0.6019 | 0.6437 | 49.950 | <0.001 |
