# Supplementary material for: Increases of Chamber Height and Base Diameter Have Contrasting Effects on Grazing Rate of Two Cladoceran Species: Implications for Microcosm Studies
Source: PLoS One. 2015 Aug 14;10(8):e0135786. doi: 10.1371/journal.pone.0135786 (PMC4537195; doi:10.1371/journal.pone.0135786)
Supplement: S3 Table — (DOCX) [file pone.0135786.s003.docx]

**Table S3** Linear regressions between the ratio of chamber height to base diameter and specific swimming activity in two *Chlorella vulgaris* -grazer systems.

|  |  | **n** | **df** | **Regression equation** | **R^2^** | **F** | **P** |
| --- | --- | --- | --- | --- | --- | --- | --- |
| ***Chlorella -Daphnia*** | Duration of quiescence (s) | 30 | (1,28) | y = 32.578x + 15.1 | 0.5832 | 39.193 | <0.001 |
|  | Duration of horizontal swimming (s) | 30 | (1,28) | y = -104.29x + 147.1 | 0.8288 | 135.556 | <0.001 |
|  | Duration of upward swimming (s) | 30 | (1,28) | y = 71.077x + 32.814 | 0.6604 | 54.451 | <0.001 |
|  | Duration of downward swimming (s) | 30 | (1,28) | y = 0.1893x + 20.294 | 0.004 | 0.000 | 0.982 |
|  | Time ratio of vertical to horizontal swimming | 30 | (1,28) | y = 2.2834x - 0.0795 | 0.8205 | 127.983 | <0.001 |
|  | Horizontal velocity (mm s^-1^) | 30 | (1,28) | y = -1.2093x + 2.3609 | 0.5902 | 40.513 | <0.001 |
|  | Upward velocity (mm s^-1^) | 30 | (1,28) | y = 0.1175x + 1.1964 | 0.0121 | 0.321 | 0.576 |
|  | Downward velocity (mm s^-1^) | 30 | (1,28) | y = -0.2042x + 1.7901 | 0.0377 | 1.116 | 0.300 |
|  | Average swimming velocity (mm s^-1^) | 30 | (1,28) | y = -0.7728x + 1.5788 | 0.5407 | 32.753 | <0.001 |
|  | Grazing rate (mL individual^-1^ h^-1^) | 30 | (1,28) | y = -0.5201x + 0.7221 | 0.7311 | 76.247 | <0.001 |
| ***Chlorella -Moina*** | Duration of quiescence (s) | 30 | (1,28) | y = 41.58x + 14.511 | 0.5614 | 35.843 | <0.001 |
|  | Duration of horizontal swimming (s) | 30 | (1,28) | y = -102.63x + 170.05 | 0.5124 | 29.425 | <0.001 |
|  | Duration of upward swimming (s) | 30 | (1,28) | y = 48.222x + 12.406 | 0.6722 | 57.417 | <0.001 |
|  | Duration of downward swimming (s) | 30 | (1,28) | y = 14.542x + 8.8893 | 0.0294 | 0.847 | 0.365 |
|  | Time ratio of vertical to horizontal swimming | 30 | (1,28) | y = 1.2567x - 0.0572 | 0.1975 | 6.891 | <0.05 |
|  | Horizontal velocity (mm s^-1^) | 30 | (1,28) | y = -1.7475x + 3.3463 | 0.5449 | 33.672 | <0.001 |
|  | Upward velocity (mm s^-1^) | 30 | (1,28) | y = 0.6637x + 1.159 | 0.087 | 2.682 | 0.113 |
|  | Downward velocity (mm s^-1^) | 30 | (1,28) | y = -0.0124x + 2.1969 | 0.004 | 0.000 | 0.983 |
|  | Average swimming velocity (mm s^-1^) | 30 | (1,28) | y = -1.344x + 2.3282 | 0.3289 | 13.464 | <0.01 |
|  | Grazing rate (mL individual^-1^ h^-1^) | 30 | (1,28) | y = -0.5769x + 0.7182 | 0.6933 | 64.436 | <0.001 |
